# Tropism Profiling of Lentiviral Vector Pseudotypes in Diverse Brain Tumor Models

**DOI:** 10.3390/pharmaceutics18010137

**Published:** 2026-01-22

**Authors:** Johannes K. Andersen, Lars A. R. Ystaas, Rolf Bjerkvig, Hrvoje Miletic, Jubayer A. Hossain

**Affiliations:** 1 Department of Biomedicine, University of Bergen, 5009 Bergen, Norway; 2 Centre for Precision Psychiatry, Institute of Clinical Medicine, University of Oslo, 0372 Oslo, Norway; 3 Division of Mental Health and Addiction, Oslo University Hospital, 0372 Oslo, Norway; 4 Department of Neurosurgery, Qilu Hospital of Shandong University and Brain Science Research Institute, Shandong University, Key Laboratory of Brain Functional Remodeling, Jinan 250062, China; 5 Department of Pathology, Haukeland University Hospital, 5009 Bergen, Norway; 6 Faculty of Nursing and Health Sciences, Nord University, 7804 Namsos, Norway

**Keywords:** lentiviral vector, gene therapy, brain tumor, GBM, DIPG, medulloblastoma, brain metastases, LCMV, VSV, rabies virus

## Abstract

**Background**: Lentiviral vectors (LVs) show promise as gene therapy tools for brain tumors, but optimal envelope protein choices for different tumor types have not been determined. **Methodology**: This study evaluated three pseudotyped LV variants—VSV-GP, FuG-B2, and LCMV-GP—across diverse brain tumor cell lines including glioblastoma (GBM), diffuse intrinsic pontine glioma (DIPG), medulloblastoma, and metastatic brain cancers. **Results**: VSV-GP and FuG-B2 pseudotypes significantly outperformed LCMV-GP across most tumor types. Both VSV-GP and FuG-B2 demonstrated high transduction efficiency in GBM and DIPG cells, though some cell lines displayed selective preferences for one pseudotype over the other. Medulloblastoma cells were challenging to transduce, with only VSV-GP achieving substantial efficacy. Metastatic brain cancers showed distinct tropism patterns: melanoma metastases were preferentially transduced by the FuG-B2 pseudotype, while lung metastases showed preference for the VSV-GP pseudotype. **Conclusions**: These findings suggest envelope protein selection should be tailored to specific brain tumor types. VSV-GP appears most suitable for medulloblastoma and lung metastases, FuG-B2 for melanoma metastases, and both for GBM and DIPG gene therapy applications. The study provides crucial guidance for translating lentiviral gene therapy to clinical applications, supporting personalized treatment strategies based on tumor-specific vector tropism profiles.

## 1. Introduction

Developed as a spin-off from human immunodeficiency virus (HIV) research in the mid-1990s, LVs have emerged as one of the most commonly used gene therapy vectors [1,2]. These replication-defective vectors offer several key features that make them excellent vectors for gene therapy [3]. Firstly, LVs have the ability to transduce both dividing and quiescent cells, making them particularly suitable for targeting quiescent or irregularly dividing cells such as neural cells or cancer stem cells. Secondly, the integration of therapeutic genes into the host genome ensures stable, long-term gene expression, a crucial requirement for effective cancer treatment strategies [4]. Thirdly, LVs have shown a relatively safer integration profile both in terms of carcinogenicity and immunogenicity [1,5,6,7]. Finally, LVs are amenable to pseudotyping with different envelope proteins, which confers multiple advantages. Pseudotyping refers to the process of incorporating heterologous envelope proteins from related or distant types of viruses, potentially enhancing tropism to target cells. Apart from modulating the tropism, pseudotyping may further enhance vector stability [8,9], reduce immunogenicity [3,10,11], and, very importantly, reduce the lentiviral portion in the vector, which consequently maximizes vector biosafety. A unique combination of all these beneficial characteristics indeed facilitates the use of LVs in various disease conditions [12].

LVs have been pseudotyped with a wide variety of heterologous viral envelope proteins, each conferring distinct cellular targeting properties and transduction efficiencies [13]. Among these, envelope glycoproteins (GPs) from vesicular stomatitis virus (VSV), lymphocytic choriomeningitis virus (LCMV) [14,15], and rabies virus (RV) have shown promise in cancer gene therapy applications. VSV-GP pseudotyped vectors demonstrate broad cellular tropism and high stability [3,11], while LCMV-GP exhibits enhanced neurotropism [16]. Additionally, by swapping various regions of two different envelope proteins, it is also possible to impart useful characteristics to the resulting fusion protein. Viral envelope proteins are often divided into surface and transmembrane subunits (TM). The TM subunit has three domains: the ectodomain, transmembrane domain, and cytoplasmic tail (CT). It is mainly the surface unit and the ectodomain that dictate the tropism of the corresponding virion [17]. The CT domain is involved in interactions with the viral core and regulates the stability of the virion in several ways [18]. The fusion glycoprotein FuG-B2 was generated by replacing the CT domain of the RV glycoprotein with that of VSV to optimize transduction efficiency and targeting specificity [18,19,20,21].

The diverse tropism profiles of these pseudotyped vectors make them particularly attractive for treating heterogeneous tumor types, where different envelope proteins may demonstrate varying affinities for distinct cancer cell populations. This selectivity becomes especially critical when targeting brain tumors, which encompass a diverse group of solid neoplasms occurring throughout the central nervous system (CNS). Primary brain tumors develop in situ within the CNS and are graded by WHO on a scale of I to IV, with grade III and IV tumors being the most malignant high-grade gliomas (HGGs). Secondary brain tumors, or brain metastases, originate from cancers in other body parts and spread to the brain, commonly from lung, melanoma, colon, and breast cancers. Brain metastases are nearly 10 times more prevalent than primary brain tumors [22]. Despite decades of research, the prognosis for malignant brain tumors remains poor, with most HGGs and brain metastases being practically incurable, highlighting the urgent need for novel therapeutic approaches [23].

While VSV-GP and LCMV-GP pseudotypes of LVs have been used to deliver suicide genes in GBM tumors, manifesting remarkable transduction efficiency, very little is known about the transducing ability of the different LV pseudotypes in different brain tumors. Additionally, FuG-B2—a rationally engineered chimeric pseudotype—remains largely uncharacterized across diverse brain tumor models despite its theoretical advantages for CNS targeting. We hypothesize that matching specific envelope proteins with corresponding tumor types could potentially enhance therapeutic efficacy while reducing systemic toxicity. In this manuscript, we report testing three prominent pseudotyped LVs in a panel of a wide variety of brain tumor models to assess their transduction capabilities.

## 2. Materials and Methods

### 2.1. Cell Lines and Cell Culture

Lung (L33, L39 and L46) and melanoma metastasis (H1, H2, H3 and H10) cells (kind gifts from Prof. Frits Thorsen, University of Bergen, Bergen, Norway) and U87 (kind gift from Prof. Rolf Bjerkvig, University of Bergen, Bergen, Norway) and HEK293 (kind gift from Prof. Boris Fehse, University Clinic Eppendorf, Hamburg, Germany) cell lines were cultured in DMEM (Dulbecco’s modified eagle’s medium) supplemented with 10% bovine fetal calf serum, 5 mg/mL penicillin/streptomycin, 2 mM L-glutamine, 3,2% NEAA, and 0,02% plasmocin. D283 and D341 human medulloblastoma cell lines were obtained from ATCC (Manassas, VA, USA) and stored and handled according to the manufacturer’s recommendations (Eagles minimum essential medium supplemented with fetal bovine serum to a final concentration of 10% for D283 and 20% for D341). Human glioma cells (BG5 [24], BG7 [24], GG16 [25], NCH421K [26], S24 [27] and P3 [25]) were cultured in serum-free neurobasal medium supplemented with 2% B27, 5 mg/mL penicillin/streptomycin, 2 mM L-glutamine, and 20 ng/mL basic fibroblast growth factor (bFGF) and 20 ng/mL epidermal growth factor (EGF). P3 was cultured without EGF supplementation. DIPG 21, DIPG 36, DIPG 38, DIPG IV, DIPG XIII (kind gifts from Prof. Michelle Monje, Standford University, Stanford, CA, USA), DIPG VU10, and DIPG VU8 (kind gifts from Prof. Esther Hulleman, Vrije University Amsterdam, Amsterdam, The Netherlands) cell lines were cultured in NB and DMEM-F12 in 1:1 ratio + B27 (-A; 12587-010), supplemented with heparin (2 mg/mL), human-EGF (20 ng/mL), human-bFGF (20 ng/mL), human PDGF-AA (10 ng/m), human PDGF-BB (10 ng/mL), and 5 mg/mL penicillin/streptomycin. All cell lines were cultured at 37 °C in 5% CO_2_ atmosphere.

### 2.2. Lentiviral GFP-Virus Production

LVs were produced by using a protocol published previously [28]. Briefly, 5.6 µg GFP expressing plasmid (M107) [29] (Figure S1) was mixed with packaging plasmids Rev (2.4 µg), RRE (4.8 µg), and either one of the three GPs (4 µg LCMV-GP [16] or 1.2 µg VSV-GP [16] or 4 µg Fug-B2 [30] in 1 mL Optimem media and 40 µL HP Xtreme reagent (Roche, Basel, Switzerland). The mixture was incubated for 20 min before transfection of HEK293 cells (ATCC # CRL-11268). LVs were harvested two times after 30 and 48 h of transfection. Harvested media was concentrated and titered by using a protocol published previously [28]. Briefly, functional titers were determined by transducing U87 with each vector preparation and the percentage of GFP-positive cells was determined after 96 h, by using flow cytometry. Functional titers were calculated based on the dilution yielding 5–20% transduction efficiency.

### 2.3. Transduction

48-well plates were pre-treated with 180 µL of poly-L-lysin. After the cells were counted using Trypan Blue and a hemocytometer, 20,000 cells were seeded in 200 µL media in 48-well plates. After 8 h of incubation, cells were transduced with different pseudotypes at MOI (multiplicity of infection) 0.25, 1 and 3 by spinoculation (centrifugation at 720× *g* at 31 °C for 1.5 h. Transduced cells were then incubated at 37 °C in 5% CO_2_ atmosphere for 96 h.

### 2.4. Flow Cytometry

Then, 96 h after transduction, the media were removed, and the cells were washed with PBS. A total of 100 µL trypsin was added to all wells and incubated for 3 min at 37 °C. A total of 150 µL of DMEM was added, and cells were collected in Eppendorf tubes. Transduction was analyzed using the BD Accuri™ C6 Plus Flow Cytometer (BD Biosciences, Aalst, Belgium). Live cells (SSCA/FSC-A) were gated, followed by single cells (FSC-H/FSC-A), and finally, gating FLA for GFP-positive cells.

### 2.5. Statistical Analysis

To analyze the proportion of cells transduced, a generalized linear mixed model was fit to the data using a beta distribution with a logit transformation. Fixed parameters included the main effects of MOI, virus, and cell type, as well as their interactions. We also performed an aggregated analysis, where specific cell types were gathered into more general tissue types. Assay date was included as a random effect in all models. Analyses were performed in R (Rstudio 2025.05.1 Build 513), using the glmmTMB package (version 1.1.11). Hypothesis tests were performed via marginal means using the emmeans package (version 1.11.1). Multiple comparisons adjustment was performed using the Tukey method.

## 3. Results

### 3.1. VSV, FuG-B2, and LCMV-GP Pseudotypes Show Strong Tropism Towards GBM Cells

**Figure 1:**
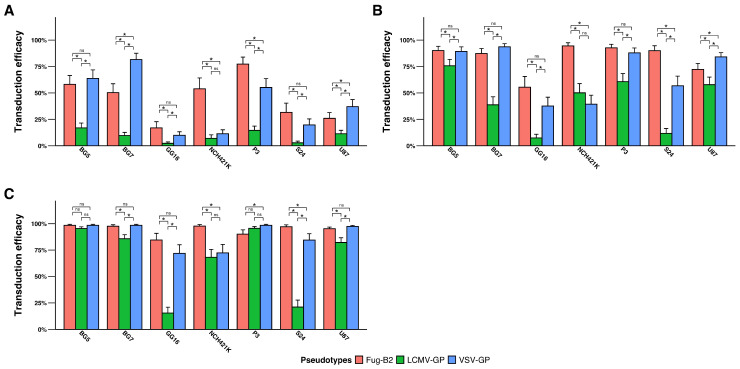
Transduction efficiency of VSV-GP, FuG-B2, and LCMV-GP pseudotyped lentiviral vectors in glioblastoma cell lines. Seven GBM cell lines were transduced at (**A**) MOI 0.25, (**B**) MOI 1, and (**C**) MOI 3, demonstrating differential pseudotype preferences. Transduction efficiency is expressed as a percentage of GFP-positive cells. Data represent mean ± SD from three independent biological replicates (*n* = 3). Statistical significance determined by generalized linear mixed models with Tukey’s multiple comparisons adjustment (* *p* < 0.05; ns, not significant).

We tested the tropism of three pseudotyped lentiviral vectors systematically in seven different GBM cell lines (Figure S1). All vectors performed well in most GBM cells (Figure 1A–C). VSV-GP and FuG-B2 pseudotypes, however, showed significantly higher transduction than LCMV-GP at all three MOIs tested (MOI 0.25, 1, and 3). At MOI 1, these vectors achieved at least 25% transduction in all 7 cell lines (Figure 1B). Interestingly, preferential transduction has also been observed in some cell lines, especially at the lowest MOI tested (Figure 1A). For instance, the VSV-GP pseudotype showed higher transduction efficacy for BG7 and U87 compared to the FuG-B2 pseudotype. However, P3 and NCH421K GBM lines were transduced more efficiently by the FuG-B2 pseudotype. At high MOIs, the transduction capability was increased substantially with all pseudotypes, indicating that GBM cells are inherently receptive to these pseudotypes. In conclusion, both VSV-GP and FuG-B2 pseudotypes transduce GBM cells more efficiently compared to the LCMV-GP pseudotype.

**Figure 2:**
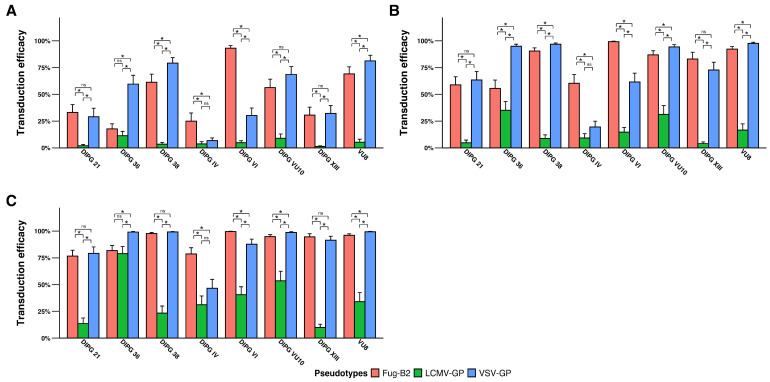
Transduction efficiency of pseudotyped lentiviral vectors in diffuse intrinsic pontine glioma cell lines. Five DIPG cell lines were transduced at three MOIs (**A**) MOI 0.25, (**B**) MOI 1, and (**C**) MOI 3, showing cell line-specific tropism patterns with VSV-GP and FuG-B2 significantly outperforming LCMV-GP. Transduction efficiency is expressed as a percentage of GFP-positive cells. Data represent mean ± SD from three independent biological replicates (*n* = 3). Statistical significance determined by generalized linear mixed models with Tukey’s multiple comparisons adjustment (* *p* < 0.05; ns, not significant).

Both VSV-GP and FuG-B2 pseudotyped lentiviral vectors exhibited robust transduction efficiency across the panel of DIPG cell lines tested, significantly outperforming the LCMV-GP pseudotype (Figure 2A–C). Comparative analysis between VSV-GP and FuG-B2 pseudotypes revealed cell line-specific tropism patterns analogous to those observed in GBM cells. At lower MOIs (MOI 0.25 and 1), the VSV-GP pseudotype demonstrated significantly enhanced transduction in DIPG 36, DIPG 38, and VU8 cell lines (Figure 2A,B), with particularly pronounced tropism observed in DIPG 36 compared to the other pseudotypes. Conversely, the FuG-B2 pseudotype exhibited preferential transduction efficiency in DIPG VI cells at these lower MOIs. Notably, in contrast to GBM cells, the majority of DIPG cell lines displayed markedly reduced susceptibility to LCMV-GP-pseudotyped LVs, even at the highest MOI tested (Figure 2C). These findings demonstrate that while VSV-GP and FuG-B2 pseudotypes both serve as effective transduction vehicles for DIPG cells, individual cell lines exhibit distinct pseudotype preferences, suggesting that envelope protein selection may be optimized based on specific DIPG molecular subtypes.

### 3.2. The VSV-GP Pseudotype Transduces Medulloblastoma Cells Effectively

**Figure 3:**
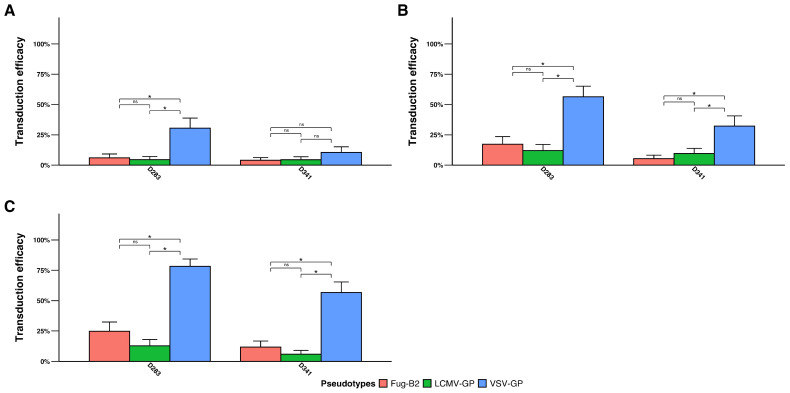
Transduction efficiency of pseudotyped lentiviral vectors in medulloblastoma cell lines. Two medulloblastoma cell lines (D283 and D341) were transduced at (**A**) MOI 0.25, (**B**) MOI 1, and (**C**) MOI 3, demonstrating VSV-GP superiority over FuG-B2 and LCMV-GP pseudotypes. Transduction efficiency is expressed as a percentage of GFP-positive cells. Data represent mean ± SD from three independent biological replicates (*n* = 3). Statistical significance determined by generalized linear mixed models with Tukey’s multiple comparisons adjustment (* *p* < 0.05; ns, not significant).

We assessed lentiviral vector tropism in two medulloblastoma cell lines: D283 and D341. Unlike GBM and DIPG cells, medulloblastoma cells showed limited transduction (Figure 3A–C). At MOI 0.25, FuG-B2 and LCMV-GP pseudotypes failed to transduce either cell line, while VSV-GP demonstrated modest but significant transduction (Figure 3A). At MOI 1, VSV-GP significantly outperformed both FuG-B2 and LCMV-GP pseudotypes in both cell lines (Figure 3B). Notably, increasing MOI to 3 did not substantially improve FuG-B2 and LCMV-GP efficiency, while VSV-GP maintained superior performance in both D283 and D341 cells (Figure 3C). These findings demonstrate that medulloblastoma cells exhibit inherent resistance to lentiviral transduction compared to other high-grade gliomas, with VSV-GP emerging as the most effective pseudotype for targeting these tumors.

### 3.3. Variable Tropism of the VSV-GP and FuG-B2 Pseudotypes in the Metastatic Brain Cancer Cells

**Figure 4:**
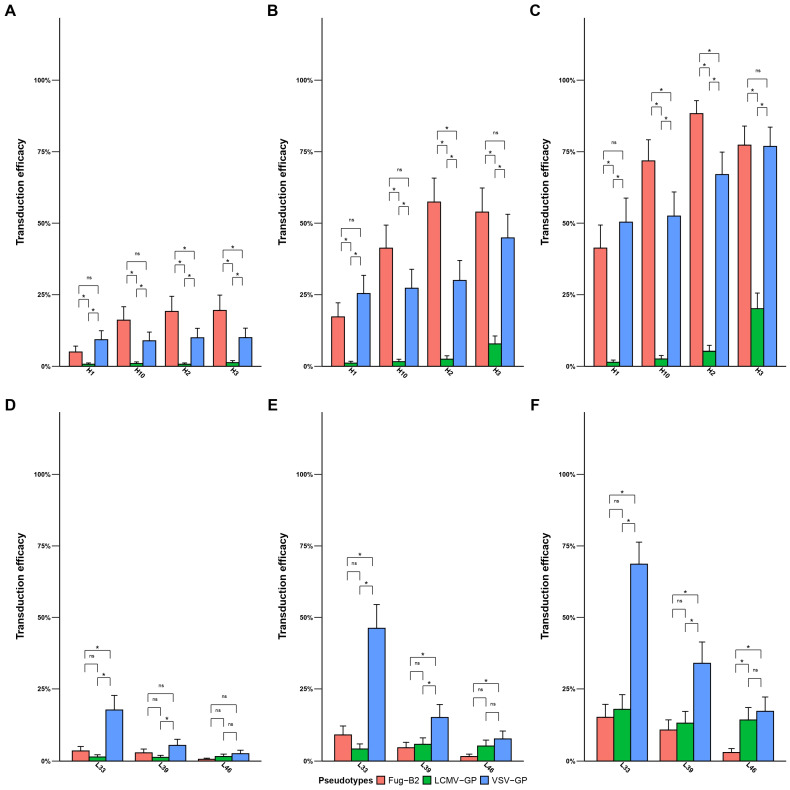
Transduction efficiency of pseudotyped lentiviral vectors in brain metastasis cell lines. Melanoma (**A**–**C**) and lung carcinoma (**D**–**F**) metastasis lines were transduced at MOI 0.25, 1, and 3, showing tumor type-specific pseudotype preferences with FuG-B2 favoring melanoma and VSV-GP superior for lung metastases. Transduction efficiency is expressed as a percentage of GFP-positive cells. Data represent mean ± SD from three independent biological replicates (*n* = 3). Statistical significance determined by generalized linear mixed models with Tukey’s multiple comparisons adjustment (* *p* < 0.05; ns, not significant).

We evaluated the transduction efficiency of the three LV pseudotypes in two major types of brain metastasis cell lines: melanoma (*n* = 4) and lung carcinoma (*n* = 3). The LCMV-GP pseudotype demonstrated minimal transduction across all metastatic cell types (Figure 4A–F). In contrast, VSV-GP and FuG-B2 pseudotypes exhibited distinct, tumor type-specific tropism patterns. In melanoma metastasis cell lines, FuG-B2 pseudotype significantly outperformed VSV-GP, transducing three out of four cell lines more efficiently at MOI 0.25 (Figure 4A), with this trend persisting at higher MOIs (Figure 4B,C). Conversely, lung metastases showed greater susceptibility to transduction by all pseudotypes (Figure 4D–F). However, among the three lung metastasis lines tested, two cell lines (L33 and L39) demonstrated clear preferential transduction by VSV-GP pseudotype over FuG-B2. These findings indicate that metastatic brain tumor cells present a more challenging transduction profile compared to primary brain tumors and exhibit pronounced pseudotype preferences, with FuG-B2 favoring melanoma metastases and VSV-GP showing superior performance in lung carcinoma metastases.

## 4. Discussion

LVs are one of the most popular viral vectors currently in use for gene therapy [2] and the safety profile of the 3rd generation self-inactivating LVs has been demonstrated by a number of clinical trials, providing confidence for its application in patients [1,2,31]. Growing evidence for the clinical safety of the LVs, combined with the preclinical success of LV-mediated gene therapy approaches in HGGs [3], highly encourage more translational studies and clinical trials with LVs. To design and perform translational studies with LVs, in this context, one important question that needs to be addressed first is which envelope protein should be used, as the transduction efficiency of different LV pseudotypes in various types of brain tumors is not known.

Our findings demonstrate that LVs represent an effective vector platform for gene therapy across multiple brain tumor types, providing further support for translational studies with this vector system. Our comparative analysis reveals that VSV-GP and FuG-B2 pseudotypes are both suitable for targeting GBM and DIPG, with VSV-GP showing preferential efficacy for medulloblastoma and lung carcinoma metastases, while FuG-B2 demonstrates superior performance in melanoma metastases. However, important limitations of this study warrant consideration. Firstly, the panel of metastatic brain tumors examined was relatively smaller compared to primary tumors (GBM and DIPGs), necessitating additional studies to establish confident comparisons between the pseudotypes in brain metastases. The observed weak transduction efficiency in lung metastases across all pseudotypes highlights the need for expanded investigation of other metastatic brain tumor types and their susceptibility to these pseudotypes. Secondly, 3D tumor architecture in clinical settings may alter the transduction capability of the vectors in relation to 2D monolayer culture that we have exclusively used.

Additionally, the broader transduction profile of VSV-GP presents context-dependent implications. For suicide gene therapy, broader tropism enhances tumor-killing capacity through bystander effect mediated by transduced normal brain cells [6]. Conversely, for immunostimulatory gene delivery, off-target transduction may necessitate regulatable expression systems to mitigate safety concerns [32].

Furthermore, future studies should elucidate whether reduced transduction efficiency stems from poor pseudotype tropism or post-entry cellular barriers. Post-entry barriers refer to the fact that host cell restriction factors can interfere with vector trafficking and expression at diverse steps following cell entry, from reverse transcription to nucleic acid translation. These barriers include intrinsic restriction factors such as TRIM5α and SAMHD1 that may be constitutively expressed in certain cell types or induced by type I interferons [33]. LCMV-GP pseudotyped lentiviral vectors achieved robust transduction in some GBM lines in our study, consistent with prior reports of efficient GBM transduction and therapeutic efficacy in xenograft models [16,34]. However, LCMV-GP showed minimal transduction in DIPG, medulloblastoma, and brain metastases, significantly underperforming compared to VSV-GP and FuG-B2. This tumor type-dependent tropism may be due to differential expression of alpha-dystroglycan, the primary LCMV-GP receptor.

Our data support the concept that envelope protein selection can be optimized for specific tumor types, potentially enhancing the therapeutic efficacy of LV-mediated gene therapy. These findings underscore the importance of systematically evaluating different LV pseudotypes across various CNS pathologies, including both neoplastic and non-neoplastic disease models, to advance current gene therapy strategies.

## Data Availability

The original contributions presented in this study are included in the article/Supplementary Material (Table S1). Further inquiries can be directed to the corresponding author.

## Supplementary Materials

The following supporting information can be downloaded at: https://www.mdpi.com/article/10.3390/pharmaceutics18010137/s1, Figure S1: Schematic overview of the study design.; Table S1: List of reagents, biological materials and equipments used.

## Abbreviations

The following abbreviations are used in this manuscript:

LV	Lentiviral Vector
GP	Glycoprotein
VSV	Vesicular Stomatitis Virus
LCMV	Lymphocytic Choriomeningitis Virus
RV	Rabies Virus
FuG-B2	Fusion Glycoprotein B2
CT	Cytoplasmic Tail
TM	Transmembrane
CNS	Central Nervous System
GBM	Glioblastoma
DIPG	Diffuse Intrinsic Pontine Glioma
HGG	High-Grade Glioma
MOI	Multiplicity of Infection
PBS	Phosphate-Buffered Saline
DMEM	Dulbecco’s Modified Eagle’s Medium
NEAA	Non-Essential Amino Acid
bFGF	Basic Fibroblast Growth Factor
EGF	Epidermal Growth Factor
PDGF-AA	Platelet-Derived Growth Factor Isoform
PDGF-BB	Platelet-Derived Growth Factor Isoform
GFP	Green Fluorescent Protein
RRE	Rev Response Element
HP	High-Performance (context: HP Xtreme reagent)
ATCC	American Type Culture Collection
WHO	World Health Organization
HIV	Human Immunodeficiency Virus
TRIM5α	Tripartite Motif-containing Protein 5 alpha
SAMHD1	SAM Domain- and HD Domain-Containing Protein 1
