# Supplementary material for: Tropism Profiling of Lentiviral Vector Pseudotypes in Diverse Brain Tumor Models
Source: Pharmaceutics. 2026 Jan 22;18(1):137. doi: 10.3390/pharmaceutics18010137 (PMC12844675; doi:10.3390/pharmaceutics18010137)
Supplement: Supplementary file 1 [file pharmaceutics-18-00137-s001.zip › pharmaceutics-4053520-supplementary.pdf]

pharmaceutics

MDPI

Tropism Profiling of Lentiviral Vector Pseudotypes in Diverse Brain Tumor Models

Johannes K. Andersen, Lars A.R. Ystaas, Rolf Bjerkvig, Hrvoje Miletic and Jubayer A. Hossain

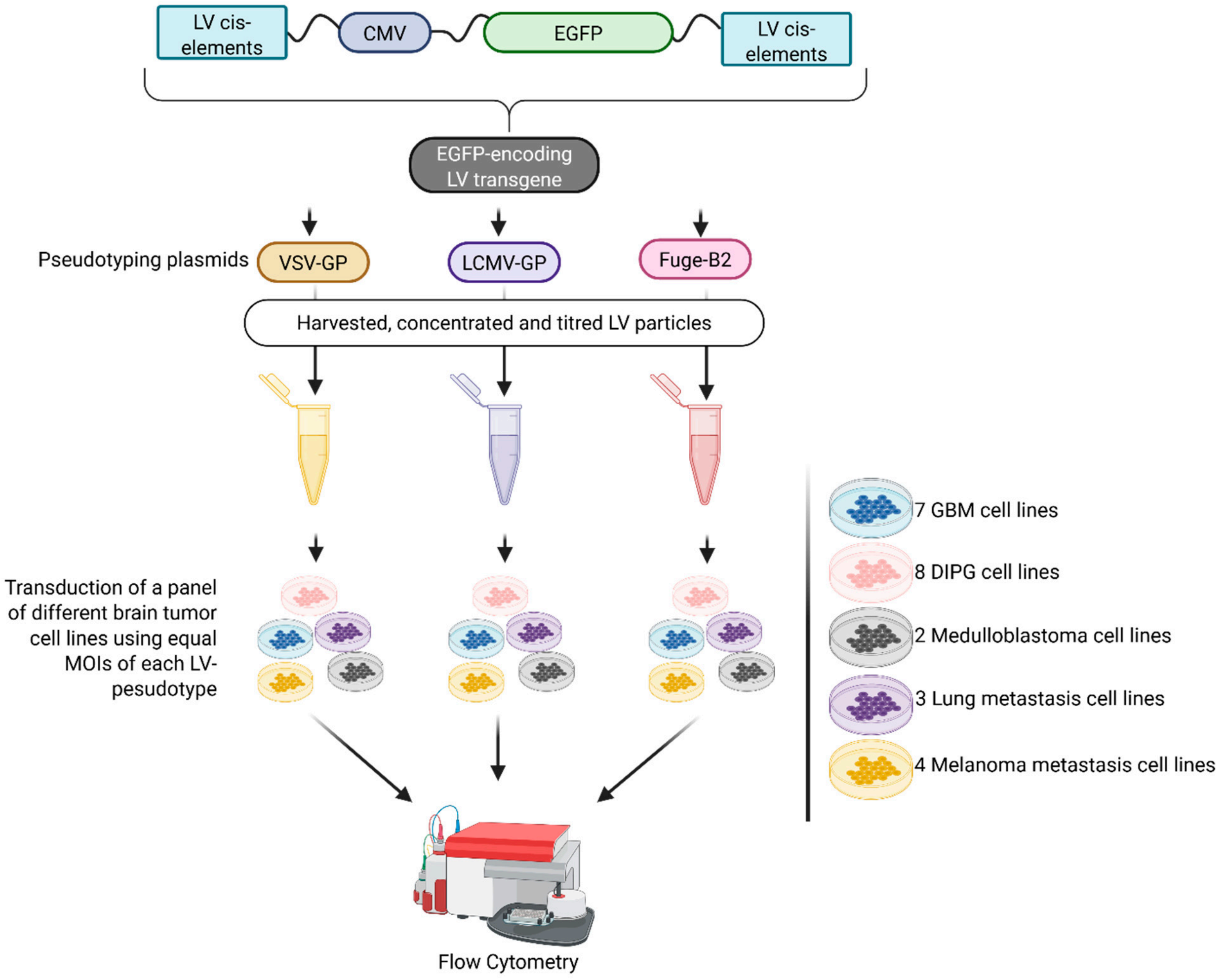

Figure S1. Schematic overview of the study design. The lentiviral transgene map is simplified; packaging plasmids (RRE, Rev) are not shown. EGFP-encoding lentiviral vectors were pseudotyped with three different envelope glycoproteins (VSV-GP, LCMV-GP, or FuG-B2). Following production, concentration, and titration, each pseudotype was used to transduce a panel of 24 brain tumor cell lines representing four tumor categories (7 GBM, 8 DIPG, 2 medulloblastoma, and 7 metastatic lines) at three MOI values (0.25, 1, and 3). Transduction efficiency was quantified by flow cytometry 96 hours post-transduction to determine pseudotype-specific tropism profiles across different brain tumor types. Figure created by using Biorender.

Table S1. List of reagents, biological materials and equipments used.

Category	Reagent/Material	Source/Supplier
Cell Lines	U87	ATCC
	HEK293	
	D283	

Category	Reagent/Material	Source/Supplier
	D341	
	BG5, BG7	See methods
	GG16	
	NCH421K	
	S24	
	P3	
	DIPG 21, 36, 38, IV, XIII	Prof. Michelle Monje
	DIPG VU10, VU8	Prof. Esther Hulleman
	L33, L39, L46	Prof. Frits Thorsen
	H1, H2, H3, H10	
Plasmids	M107 (GFP expression plasmid)	See methods
	Rev (packaging plasmid)	Addgene # 12253
	RRE (packaging plasmid)	Addgene # 12251
	LCMV-GP (envelope plasmid)	See methods
	VSV-GP (envelope plasmid)	
	FuG-B2 (envelope plasmid)	
Culture Media	DMEM	Gibco
	Eagles Minimum Essential Medium	
	Neurobasal Medium	
	DMEM-F12	
Media Supplements	Fetal Bovine Serum (FBS)	
	Bovine Fetal Calf Serum	
	Penicillin/Streptomycin	
	L-glutamine	
	NEAA	
	Plasmocin	Invivogen
	B27 supplement	Gibco
	bFGF (basic Fibroblast Growth Factor)	Peprotech
	EGF (Epidermal Growth Factor)	
	Human PDGF-AA	
	Human PDGF-BB	
	Heparin	Sigma-Aldrich
Transfection	Optimem Media	Gibco
	HP Xtreme Reagent	Roche
Cell Culture	Poly-L-lysine	Sigma-Aldrich
	Trypan Blue	
	Trypsin	Gibco
	PBS (Phosphate Buffered Saline)	Sigma-Aldrich
Equipment	BD Accuri C6 Plus Flow Cytometer	BD Biosciences
Software	R/RStudio	Open source
	glmmTMB package	R package
	emmeans package	R package
